# New Role for Photoexcited Na_2_ Eosin Y *via* the Direct Hydrogen Atom Transfer Process in Photochemical Visible-Light-Induced Synthesis of 2-Amino-4*H*-Chromene Scaffolds Under Air Atmosphere

**DOI:** 10.3389/fchem.2022.880257

**Published:** 2022-06-09

**Authors:** Farzaneh Mohamadpour

**Affiliations:** School of Engineering, Apadana Institute of Higher Education, Shiraz, Iran

**Keywords:** photoexcited Na_2_ eosin Y, photochemical synthesis, visible light mediated, 2-amino-4H-chromene scaffolds, green chemistry

## Abstract

The Knoevenagel–Michael cyclocondensation of malononitrile, aryl aldehydes, and resorcinol was used as a multicomponent green tandem strategy for the metal-free synthesis of 2-amino-4*H*-chromene scaffolds. Through a visible-light-induced process, the photo-excited state functions derived from Na_2_ eosin Y were used as direct hydrogen atom transfer catalysts in aqueous ethanol at ambient temperature. The purpose of this study was to examine the further use of an organic dye that does not contain metal and is inexpensive and commercially available. Na_2_ eosin Y is synthesized by photochemical means using the least amount of catalyst, which results in excellent yields, energy efficiency, and environmental friendliness, high atom economy, time-saving features, and ease of operation. As a result, some properties of green and sustainable chemistry are met. This kind of cyclization can be performed on a gram scale, indicating the potential utility of this reaction in industry.

## Introduction

Eosin Y is a metal-free organic dye that has gained widespread use in recent years as a cost-effective and environment friendly alternative to transition-metal-based photocatalysts (Zhu et al., 2018; Wang et al., 2019; Yan et al., 2019; Chen et al., 2020). Successfully oxidized/reduced target substrates by their incited mode in eosin Y-catalyzed photoredox reactions are typically dependent on whether the substrates’ prospective oxidability or reducibility falls within the scope of eosin Y (Yan et al., 2019). The electrochemical requirements that have been discussed have limited the range of eosin Y-catalyzed photochemical processes. Eosin Y is distinguished from other organic dyes by its distinct xanthene and phenol moieties, as well as notable acid–base properties, which can result in four distinct constructions. In most earlier reports on photoreactions, there is ample evidence that the anionic kinds of eosin Y exhibit photocatalytic properties, but the neutral types are thought to have typical inactivity and are ignorable in potentially applied synthesis procedures (Hari and König, 2014; Majek et al., 2014). The structural properties of eosin Y have inspired a team of Wang (Zhao and Wang, 2018) and Wu (Fan et al., 2018) to innovate in the discovery of novel activating states of photoexcited eosin Y in recent years. The researchers observed that incited modes derived from neutral eosin Y may operate as photoacids and direct hydrogen atom transfer (HAT) catalysts for activating glycals and native C–H bonds in the order they were identified (Yan et al., 2019).

HAT (hydrogen atom transfer) is a fundamental stage that may be responsible for a variety of chemical, environmental, and biological processes. In recent years, benzophenone- and quinone-mediated direct HAT catalysis has been promoted as a tool for activating the C–H bond under light radiation (Ravelli et al., 2016; Romero and Nicewicz, 2016; Capaldo and Ravelli, 2017). Direct HAT catalysis mediated by benzophenone and quinone has recently been established as a viable method for irradiating the C–H bond (Ravelli et al., 2016; Romero and Nicewicz, 2016; Capaldo and Ravelli, 2017). Due to the similarities between eosin Y and quinones (Ravelli et al., 2016; Romero and Nicewicz, 2016), Wu and others suggested that when exposed to visible light, eosin Y may work as a direct HAT catalyst, activating a C–H bond and creating radical species for further functionalities (Fan et al., 2018). The radical species formed from eosin Y is unlikely to suffer the kinds of side reactions seen in HAT catalysis with diaryl ketones, allowing for a reverse transfer of hydrogen atom, due to its captodative and steric features. Wu and others demonstrated that when eosin Y in the neutral state is exposed to the visible spectrum, it may successfully initiate numerous C (sp^3^)-H and C (sp^2^)-H bonds to start generating the matching carbon radicals, allowing radical introduction to multiple alkenes with electron deficit. This method covers a wide range of substrates and has a high group tolerance. The needed C–H alkylation compounds were synthesized with good yields and site selectivity. A number of C (sp^3^)-H and C (sp^2^)-H bonds of ethers, thioethers, alcohols, aldehydes, and cyclohexanes were radically alkylated with acceptable site selection (e.g., 10 c). This approach can be used to a wide range of tri- and tetrasubstituted olefins with various properties. The substrate restrictions of traditional SET-based redox reactions are circumvented by this HAT catalysis technique (Yan et al., 2019).

In the eco-friendly synthesis of organic molecules, visible light irradiation has also proven to be a trustworthy strategy for green chemists because of its abundant energy reserves, low prices, and renewable source of energy (Mohamadpour, 2020; Mohamadpour, 2021a). In general, visible light sources such as light emitting diodes and tiny fluorescent lamps are used for various conversions.

Because of their biological actions, chromenes and their equivalents have gotten a lot of attention such as antimicrobial (Kathrotiya and Patel, 2012), antifungal (Alvey et al., 2009), anti-inflammatory (Moon et al., 2007), antibacterial (Kumar et al., 2009), antioxidant (Symeonidis et al., 2009), antileishmanial (Narender et al., 2004), anti-HIV (Flavin et al., 1996; Rueping et al., 2008), anticancer (Abdelrazek et al., 2004; Paliwal et al., 2013), and hypotensive (Cai et al., 2009). Also, they are used as inhibitors (Wang et al., 2000; Huynh et al., 2012).

Several multicomponent reactions for manufacturing 2-amino-4*H*-chromene scaffolds have been described against various catalysts such as glycine (Datta and Pasha, 2012), mesolite (Pawar et al., 2018), potassium phthalimide (Dekamin and Eslami, 2014), MgFe_2_O_4_NPs (Eshtehardian et al., 2020), POM@Dy-PDA (Hosseinzadeh-Baghan et al., 2020), P4VPy-CuI (Albadi and Mansournezhad, 2016), nanozeolite clinoptilolite (Baghbanian et al., 2013), water extract of lemon fruit shell ash (WELFSA) (Kantharaju and Khatavi, 2018), tungstic acid functionalized SBA-15 (Kundu et al., 2013), MIL-101(Cr)-SO_3_H (Saikia and Saikia, 2016) [Et_2_NH(CH_2_)_2_CO_2_H][AcO] (Shaikh et al., 2019), {[4,4′-BPyH][C(CN)_3_]_2_} (Zolfigol et al., 2016), DBU (Raghuvanshi and Singh, 2010), and hydrotalcite (Kale et al., 2013). Several cases arose from these surgeries. Some synthetic policies, however, include restrictions on the use of metal catalysts, harsh reaction conditions, expensive reagents, monotonous workup processes, unacceptable yields, long reaction times, environmental hazards, and the use of homogeneous catalysts that are problematically detached from the reaction mixture.

Given the foregoing factors and our interest in producing 2-amino-4*H*-chromenes, the key goal was to investigate the photocatalyst (Mohamadpour, 2021b; Mohamadpour, 2021c) under green conditions for the appropriate synthesis of these heterocyclic compounds. This study paves the new role for further usage of a metal-free organic dye with commercial availability and inexpensiveness, Na_2_ eosin Y in aforementioned photochemical synthesis. Evidence suggests that the photoexcited states of Na_2_ eosin Y act as a direct hydrogen atom transfer (HAT) catalyst in the photochemical synthesis of 2-amino-4*H*-chromenes *via* the Knoevenagel–Michael cyclocondensation reaction of aryl aldehydes, malononitrile, and resorcinol in aqueous ethanol at ambient temperature under air atmosphere. This is a successful one-pot reaction that uses extremely effective, moderate, and simple reaction conditions.

## Experimental

### Producing 4a-w

Under white light (LED) irradiation (18 W), Na_2_ eosin Y (0.5 mol%) was added to a mixture of aryl aldehydes, malononitrile, and resorcinol in an H_2_O/EtOH (2:1) (3 ml) (Supplementary Figure S2). At rt, the mixture was agitated, and TLC was used to track the reaction’s progress. The resulting solid was filtered and rinsed with H_2_O before the reaction was completed. The pure substance was then recrystallized crude solid from ethanol with no further purification. After that, the goods were classified by comparing the spectroscopic data (^1^HNMR). The spectroscopic data listed below can be found in the Supplementary Material file.

## Results and Discussion

**SCHEME 1:**
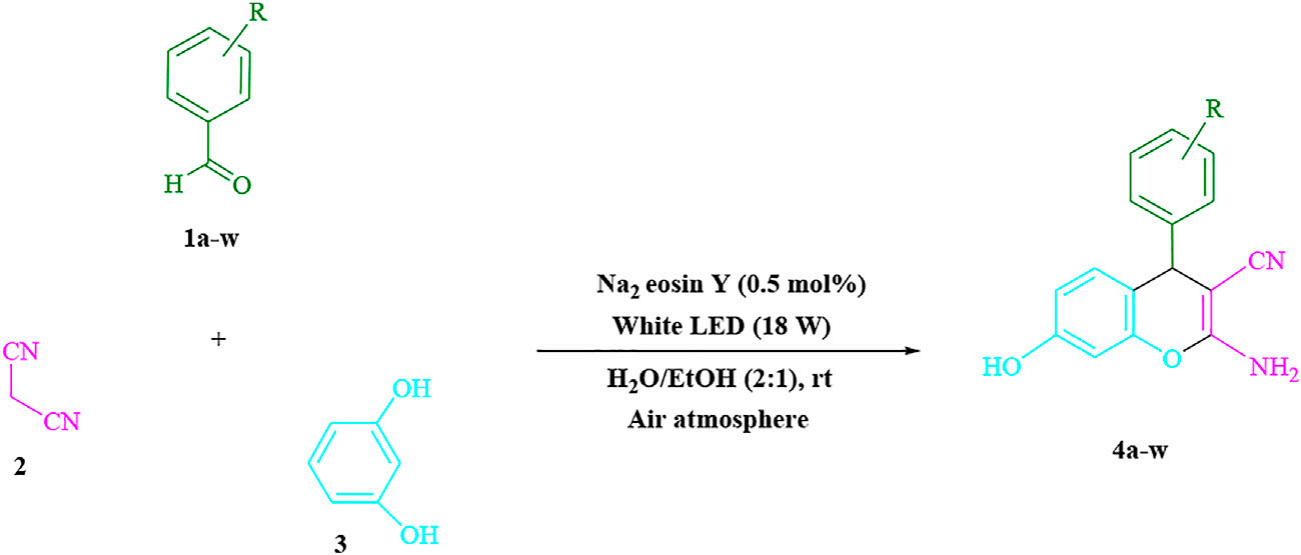
Synthesis of 2-amino-4*H*-chromene scaffolds.

To prepare **4a** in H_2_O/EtOH (2:1) (3 ml) at ambient temperature under LED irradiation, the reaction between malononitrile, benzaldehyde, and resorcinol was studied first. In 3 ml of H_2_O/EtOH (2:1) for 15 min with no photocatalysts, there was a 57% of **4a** at rt. Various organic photocatalysts, such as Na_2_ eosin Y, erythrosin B, phenanthrenequinone, rhodamine B, acenaphthenequinone, riboflavin,9*H*-xanthen-9-one, fluorescein, and rose bengal, were tested in similar settings to stimulate the process. While achieving the matching product **4a**, the progression of this reaction was seen in 51–93% yields. The results showed that in such a response, Na_2_ eosin Y performed better. The yield was enhanced to 93% by using 0.5 mol% Na_2_ eosin Y. (Table 1, entry 3). In addition, CHCl_3_, toluene, THF, DMSO, CH_2_Cl_2_, CH_3_CN, and DMF, all had reduced product yields. The reaction rate and yield were enhanced by performing the reaction in EtOAc, MeOH, EtOH, H_2_O/EtOH, H_2_O, and solvent-free. The reaction went well in a 2:1 mixture of H_2_O and EtOH. Table 1 shows that in identical conditions, a yield of 93% was attained (entry 3). Different light sources were employed to screen the yield, demonstrating the effect of white light. There was a minuscule of **4a** without using the light source, according to the test control. To create product **4a** successfully, visible light and Na_2_ eosin Y are required, according to the findings. Furthermore, the increased settings were specified by irradiating white LEDs of varied intensities (10, 12, 18, and 20 W). The best results were obtained under the irradiation of white LEDs (18 W), according to Table 1 (entry 3). It was discovered that the process may be used on a variety of substrates (Table 2; Scheme 1). (More data are provided in Supplementary Table S1 in the Supplementary Material file).

**TABLE 1:** Optimization table of photocatalyst for the synthesis of **4a** ^a^.

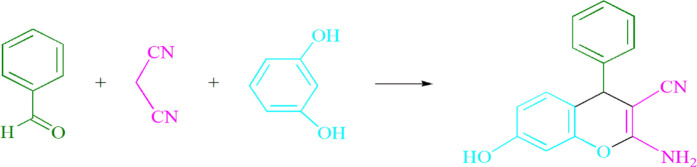					
**Entry**	**Photocatalyst**	**Light source**	**Solvent (3 ml)**	**Time (min)**	**Isolated yields (%)**
1	—	White light (18 W)	H_2_O/EtOH (2:1)	15	57
2	Na_2_ eosin Y (0.2 mol%)	White light (18 W)	H_2_O/EtOH (2:1)	5	78
**3**	**Na**_**2**_ **eosin Y (0.5 mol%)**	**White light (18 W)**	**H**_**2**_ **O/EtOH (2:1)**	**5**	**93**
4	Na_2_ eosin Y (1 mol%)	White light (18 W)	H_2_O/EtOH (2:1)	5	93
5	Erythrosin B (0.5 mol%)	White light (18 W)	H_2_O/EtOH (2:1)	5	51
6	Phenanthrenequinone (0.5 mol%)	White light (18 W)	H_2_O/EtOH (2:1)	5	53
7	Rhodamine B (0.5 mol%)	White light (18 W)	H_2_O/EtOH (2:1)	5	72
8	Acenaphthenequinone (0.5 mol%)	White light (18 W)	H_2_O/EtOH (2:1)	5	57
9	Riboflavin (0.5 mol%)	White light (18 W)	H_2_O/EtOH (2:1)	5	64
10	9*H*-xanthen-9-one (0.5 mol%)	White light (18 W)	H_2_O/EtOH (2:1)	5	60
11	Fluorescein (0.5 mol%)	White light (18 W)	H_2_O/EtOH (2:1)	5	77
12	Rose bengal (0.5 mol%)	White light (18 W)	H_2_O/EtOH (2:1)	5	68

a Reaction conditions: benzaldehyde (1 mmol), malononitrile (1 mmol), resorcinol (1 mmol) in H_2_O/EtOH (2:1) (3 ml), white LED (18 W), and various photocatalysts at rt. Bold values provides optimal conditions for reaction.

**TABLE 2:** Photoexcited Na_2_ eosin Y as a photocatalyst for the synthesis of 2-amino-4*H*-chromene scaffolds.

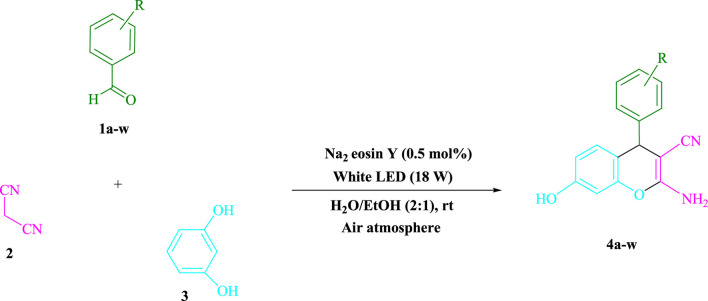		
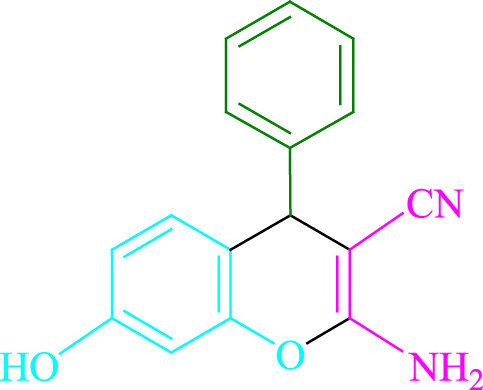 **4a** (5min, 93%)MP. 234‐236°CLit. 232‐234°C [27]	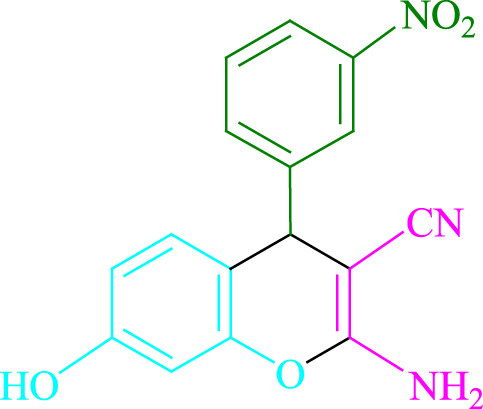 **4b** (5min, 94%)Mp. 166‐168°CLit. 168‐170°C [32]	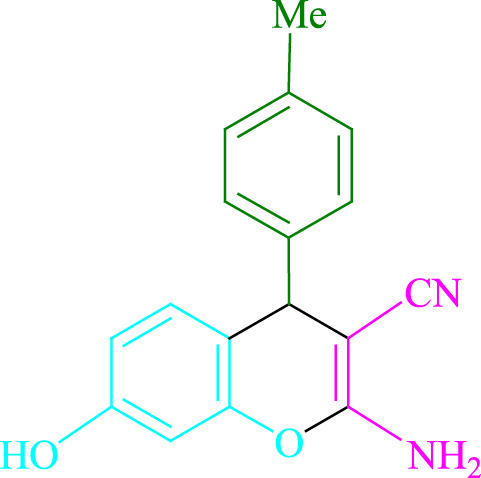 **4c** (3min, 91%)Mp. 185‐187°CLit. 186‐188°C [33]
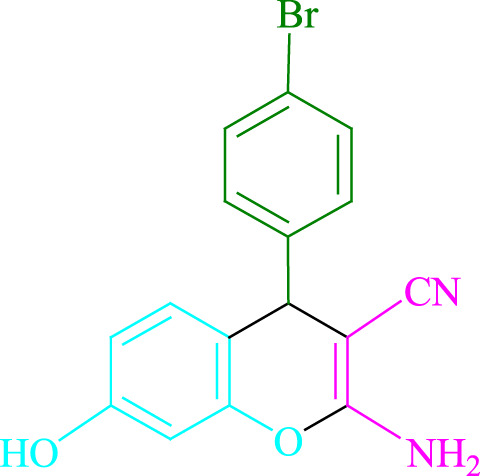 **4d** (10min, 88%)Mp. 223‐225°CLit. 222‐224°C [24]	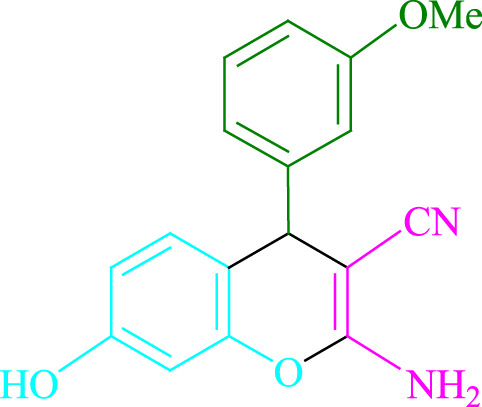 **4e** (7min, 91%)Mp. 179‐181°CLit. 180‐182°C [33]	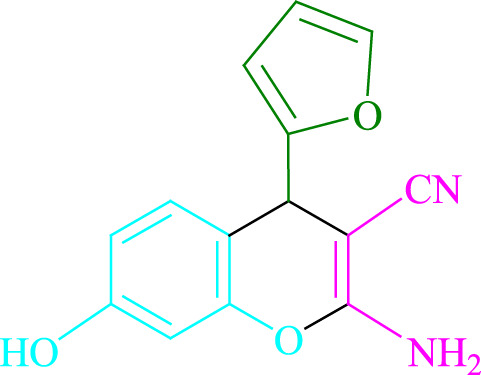 **4f** (5min, 95%)Mp. 192‐194°CLit. 190‐192°C [32]
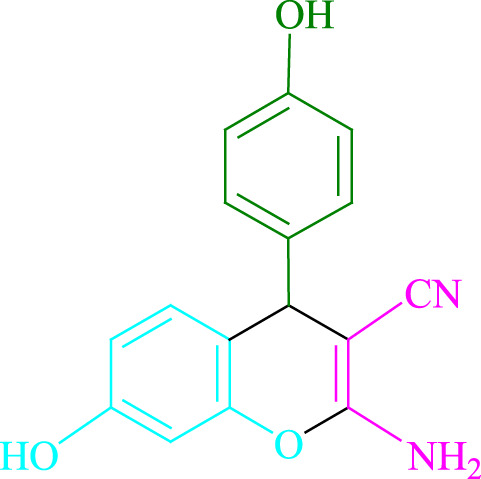 **4g** (10min, 84%)Mp. 249‐251°CLit. 250‐252°C [23]	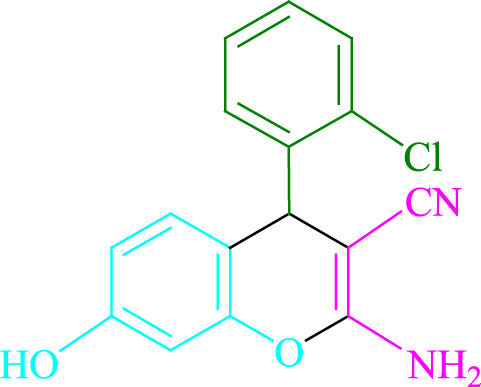 **4h** (5min, 86%)Mp. 187‐189°CLit. 189‐191°C [27]	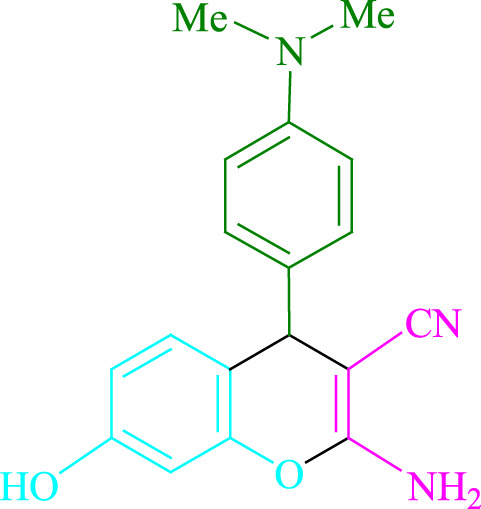 **4i** (5min, 92%)Mp. 194‐196°CLit. 194‐196°C [24]
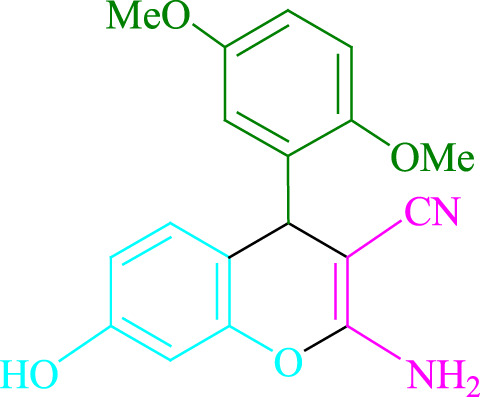 **4j** (9min, 87%)Mp. 200‐202°CLit. 198‐200°C [35]	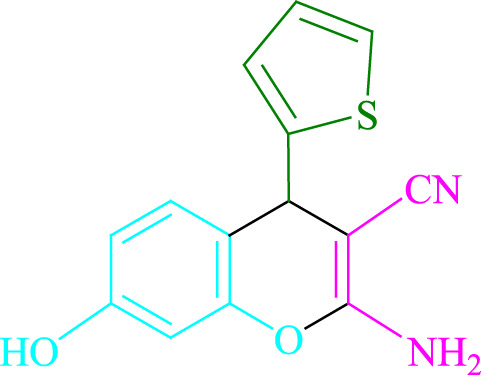 **4k** (5min, 92%)Mp. 211‐213°CLit. 210‐212°C [24]	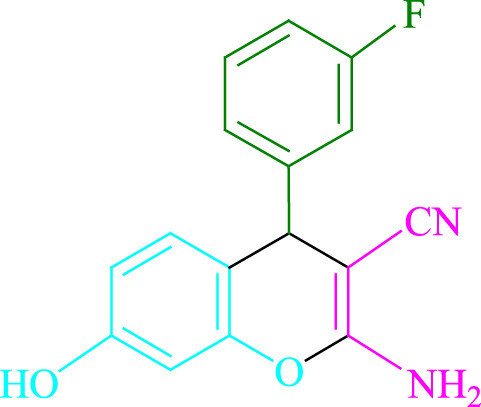 **4l** (3min, 93%)Mp. 146‐148°CLit. 148‐150°C [29]
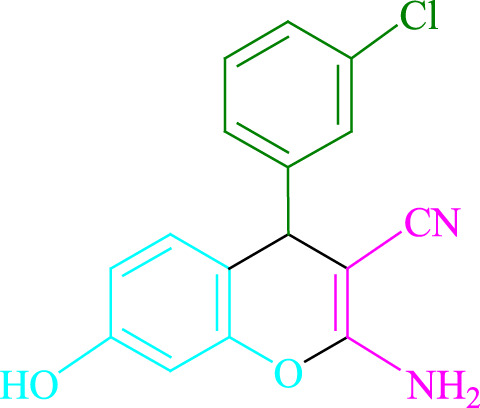 **4m** (7min, 89%)Mp. 175‐177°CLit. 176‐178°C [35]	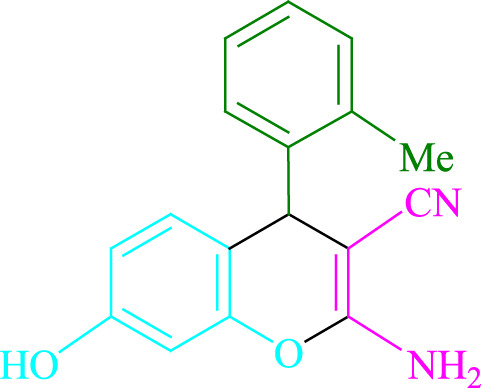 **4n** (3min, 94%)Mp. 229‐229°CLit. 223‐231°C [25]	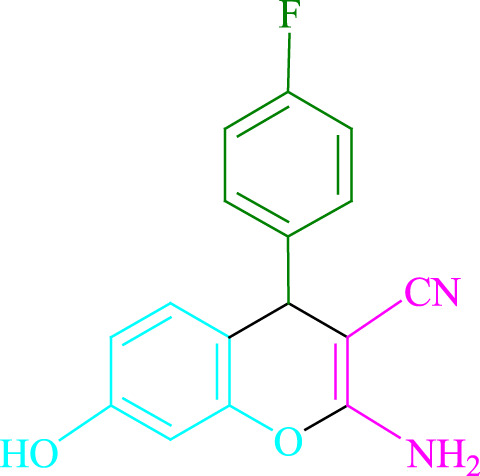 **4o** (3min, 96%)Mp. 189‐191°CLit. 188‐190°C [27]
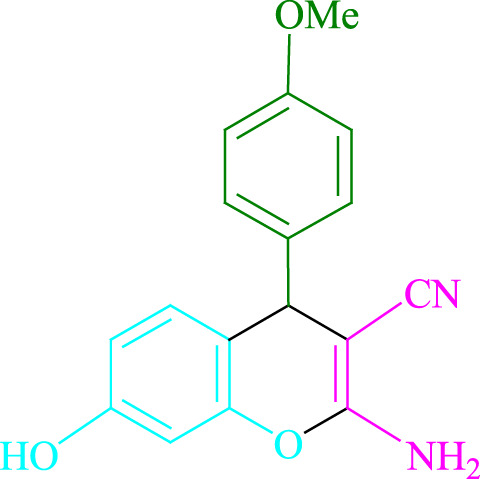 **4p** (7min, 88%)Mp. 208‐210°CLit. 210‐212°C [29]	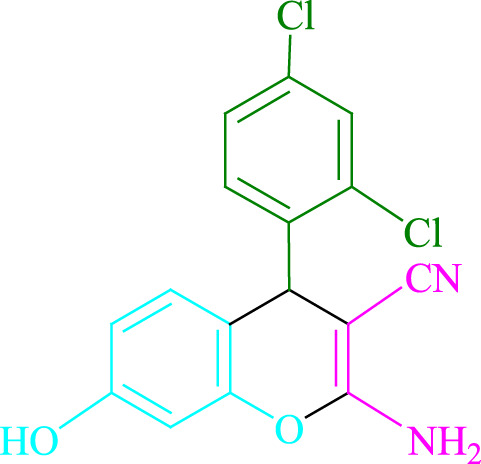 **4q** (7min, 88%)Mp. 259‐261°CLit. 257‐259°C [27]	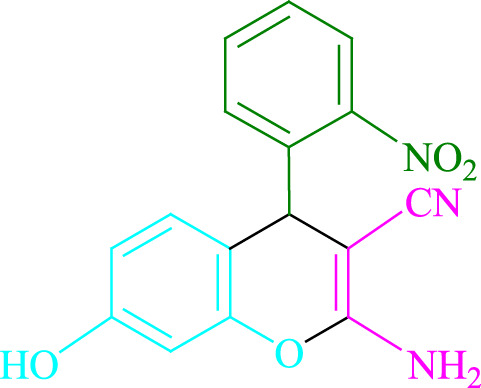 **4r** (3min, 96%)Mp. 160‐162°CLit. 162‐163°C [34]
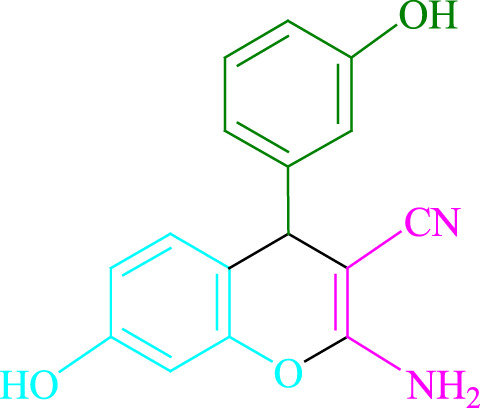 **4s** (8min, 87%)Mp. 218‐220°CLit. 219‐221°C [33]	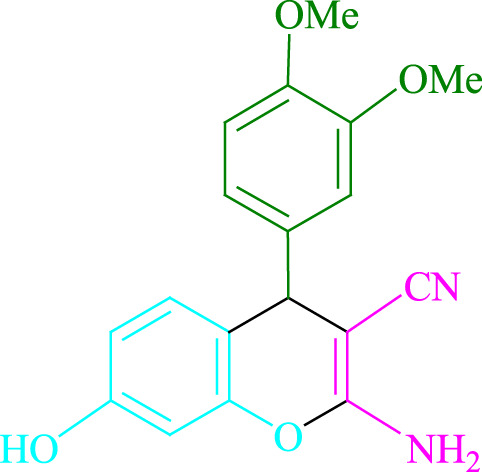 **4t** (9min, 85%)Mp. 229‐231°CLit. 227‐229°C [33]	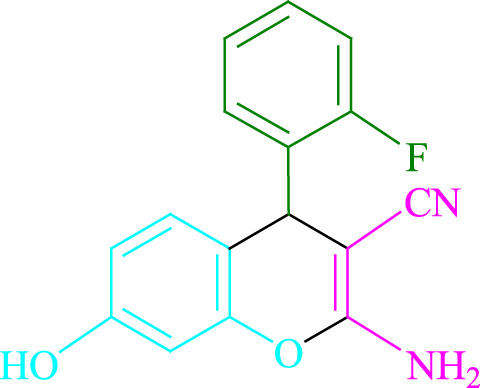 **4u** (3min, 94%)Mp. 201‐203°CLit. 200‐202°C [34]
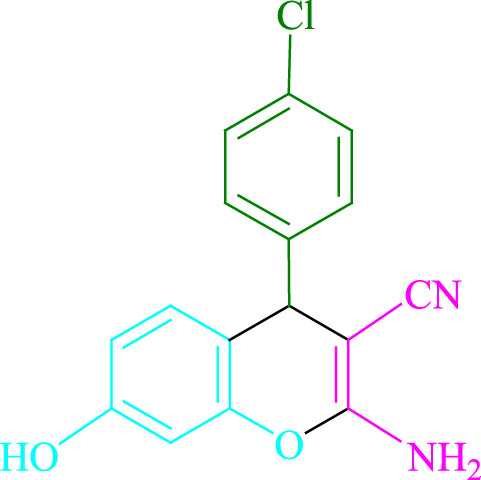 **4v** (7min, 87%)Mp. 163‐165°CLit. 162‐163°C [27]	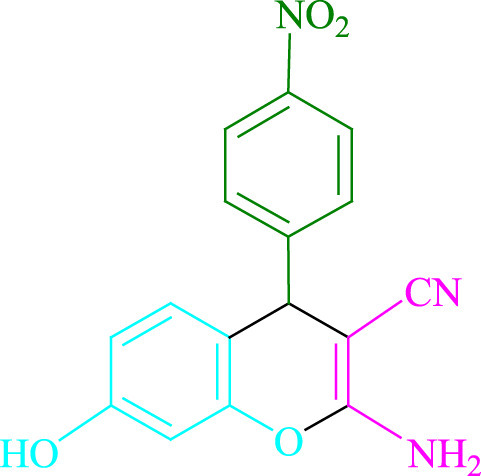	

**SCHEME 2:**
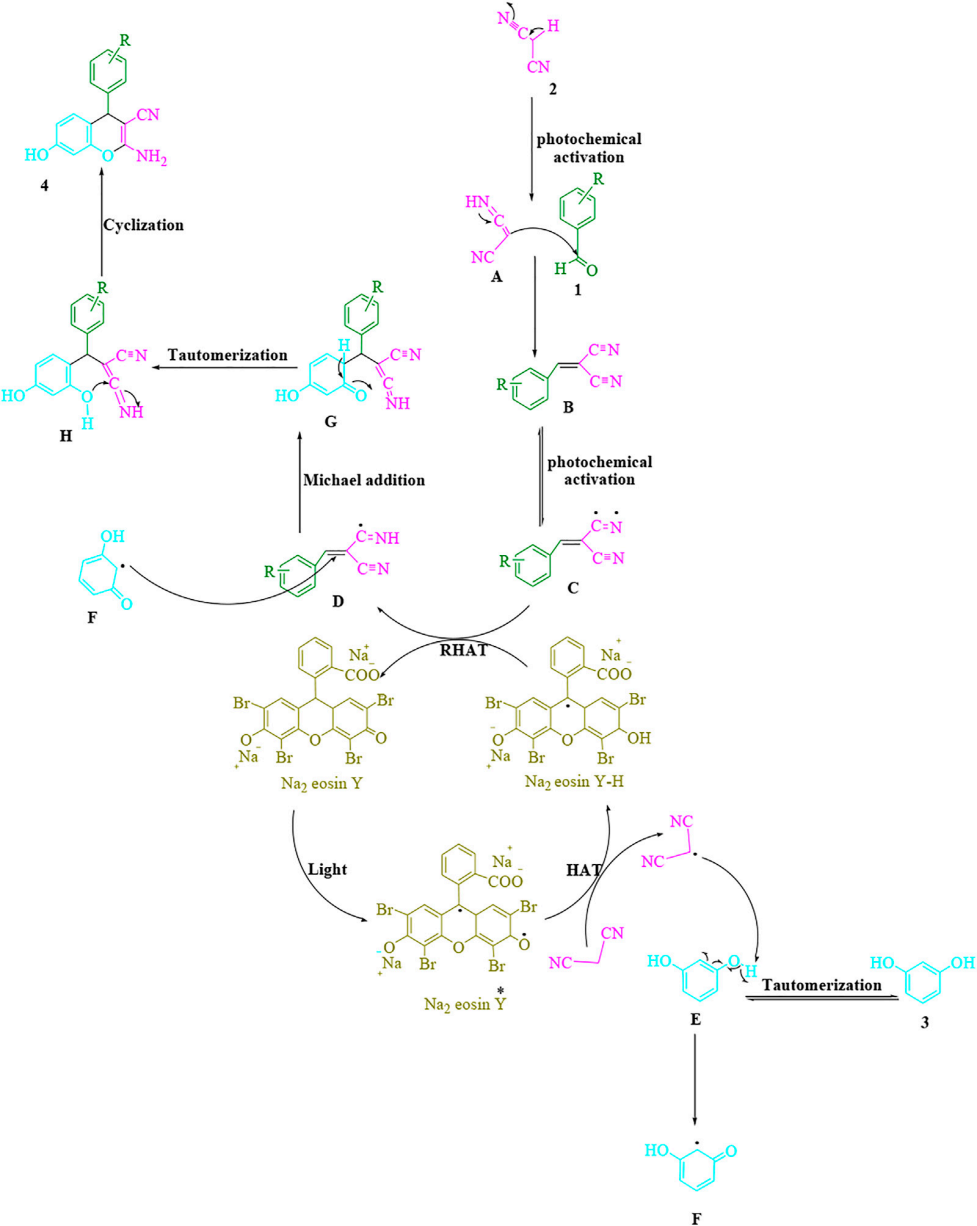
The proposed mechanistic pathway to synthesize the 2-amino-4*H*-chromene scaffolds.

The proposed technique is depicted in Scheme 2. Malononitrile (**2**) is exposed to tautomerization through the visible light to provide (**A**). Then, aldehydes (**1**) and (**A**) combine to make arylidenemalononitrile (**B**), which undergoes photochemical activation to yield a radical intermediate (**C**). The visible light can be changed in part by the application of more energy to speed up this reaction. Eosin Y-made photoexcited modes can operate as direct HAT catalysts to activate C–H bonds eosin, according to earlier findings (Fan et al., 2018; Yan et al., 2019; Chen et al., 2020). Through a HAT method, the malononitrile radical is produced to boost the visible light triggered Na_2_ eosin Y*. The reverse hydrogen atom transfer (RHAT) method between radical adduct **C** and eosin Na_2_ Y-H produces intermediate **D** and ground-state Na_2_ eosin Y. A hydrogen atom is then removed from (**E**) by the malononitrile radical, resulting in intermediate (**F**). Then, as a Michael acceptor, intermediates (**F**) and (**D**) coalesce to produce (**G**), which undergoes intramolecular cyclization and tautomerization to yield the product (**4**).

Table 3 compares the capability of some of the catalysts used in this investigation to generate 2-amino-4*H*-chromenes. It could be used for a variety of purposes, such as the use of a short-time reaction with the least amount of photocatalyst and no by-products when exposed to visible light. The multigram-scale atom-economic amazing protocol is operative because it contains the main industrial applications that achieve both good purity and excellent performance. The atomic economy was likewise carefully managed in this sense.

**TABLE 3:** Comparison between the catalytic capacity of some catalysts in this work^a^.

Entry	Catalyst	Conditions	Time/yield (%)	References
1	Glycine	H_2_O, sonication	9 min/94	Datta and Pasha, (2012)
2	Mesolite	EtOH, reflux	30 min/93	Pawar et al. (2018)
3	Potassium phthalimide	H_2_O, reflux	12 min/94	Dekamin and Eslami, (2014)
4	MgFe_2_O_4_NPs	EtOH, 65°C	12 min/74	Eshtehardian et al. (2020)
5	POM@Dy-PDA	EtOH/H_2_O, reflux	15 min/95	Hosseinzadeh-Baghan et al. (2020)
6	P4VPy-CuI	H_2_O, reflux	15 min/94	Albadi and Mansournezhad, (2016)
7	Nanozeolite clinoptilolite	H_2_O, reflux	15 min/92	Baghbanian et al. (2013)
8	WELFSA	H_2_O, rt	1.5h/88	Kantharaju and Khatavi, (2018)
9	Tungstic acid functionalized SBA-15	H_2_O, 100°C	12 min/86	Kundu et al. (2013)
10	MIL-101(Cr)-SO_3_H	H_2_O, 100°C	180 min/82	Saikia and Saikia, (2016)
11	[Et_2_NH(CH_2_)_2_CO_2_H][AcO]	Solvent free, 60°C	12 min/92	Shaikh et al. (2019)
12	{[4,4′-BPyH][C(CN)_3_]_2_}	Solvent free, 80°C	15 min/90	Zolfigol et al. (2016)
13	DBU	EtOH,MW, 50°C	3 min/94	Raghuvanshi and Singh, (2010)
14	Hydrotalcite	H_2_O, 60°C	4 h/95	Kale et al. (2013)
**15**	**Na**_**2**_ **eosin Y**	**Visible light irradiation, H**_**2**_ **O/EtOH (2:1), rt**	**5 min/93**	**This work**

a Based on the three-component reaction of benzaldehyde, malononitrile, and resorcinol. Bold values provides optimal conditions for reaction.

## Conclusion

The photo-excited state functions produced from Na_2_ eosin Y can be used to metal-free synthesis the 2-amino-4*H*-chromene scaffolds, according to the findings. This procedure is carried out in an aqueous ethanol air environment at room temperature using visible light. The most obvious benefits of this green protocol include the use of the least amount of catalyst, high yields, efficient sides of the reaction, secure reaction conditions, and a quick operation without the use of toxic solvents or catalysts. As a result, this procedure has more advantages when it comes to meeting industrial needs and environmental issues.

## Data Availability

The original contributions presented in the study are included in the article/Supplementary Material, further inquiries can be directed to the corresponding author.
