# Supplementary material for: New Role for Photoexcited Na2 Eosin Y via the Direct Hydrogen Atom Transfer Process in Photochemical Visible-Light-Induced Synthesis of 2-Amino-4H-Chromene Scaffolds Under Air Atmosphere
Source: Front Chem. 2022 Jun 9;10:880257. doi: 10.3389/fchem.2022.880257 (PMC9218595; doi:10.3389/fchem.2022.880257)
Supplement: Supplementary file 1 [file DataSheet2.pdf]

Supporting Information

New role for photoexcited Na_2 eosin Y via the direct hydrogen atom transfer (HAT) process in photochemical visible-light-induced synthesis of 2-amino-4*H*-chromene scaffolds under air atmosphere

Farzaneh Mohamadpour *

School of Engineering, Apadana Institute of Higher Education, Shiraz, Iran

* *Corresponding author. mohamadpour.f.7@gmail.com*

2-Amino-3-cyano-7-hydroxy-4-(3-nitrophenyl)-4H-chromene (4b)

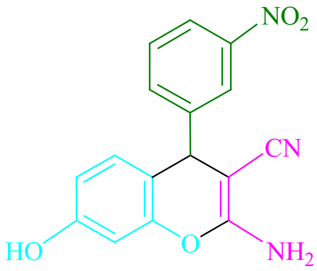

Yield: 94%; M.p. 166-168 °C; ^{1}HNMR (300 MHz, DMSO-d$_6$): 4.82 (1H, s, CHAr), 6.19 (1H, d, *J*=8.8 Hz, ArH), 6.59 (1H, d, *J*=9.6 Hz, ArH), 6.77 (1H, d, *J*=9.6 Hz, ArH), 6.97 (2H, s, NH$_2$), 7.33 (2H, d, *J*=9.6 Hz, ArH), 7.86 (2H, d, *J*=9.6 Hz, ArH), 9.69 (1H, s, OH).

2-Amino-3-cyano-7-hydroxy-4-(4-methylphenyl)-4H-chromene (4c)

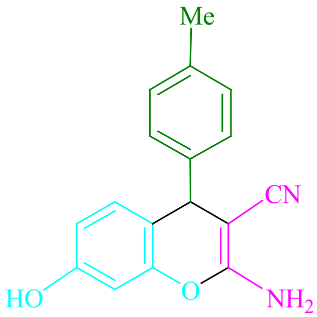

Yield: 91%; M.p. 185-187 °C; ^{1}HNMR (300 MHz, DMSO-d$_6$): 2.51 (3H, s, CH$_3$), 4.72 (1H, s, CHAr), 6.21 (1H, d, *J*=9.6 Hz, ArH), 6.70 (1H, d, *J*=9.6 Hz, ArH), 6.84 (1H, d, *J*=10.4 Hz, ArH), 7.03 (2H, s, NH$_2$), 7.17 (2H, d, *J*=9.6 Hz, ArH), 7.48 (2H, d, *J*=9.6 Hz, ArH), 9.63 (1H, s, OH).

2-Amino-3-cyano-7-hydroxy-4-(4-methoxyphenyl)-4H-chromene (4p)

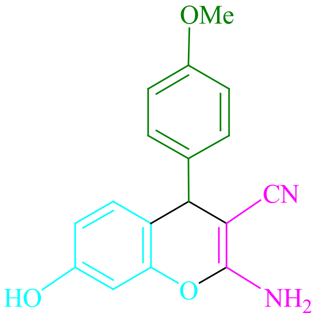

Yield: 88%; M.p. 208-210 °C; ^{1}HNMR (300 MHz, DMSO-d_6): 3.71 (3H, s, OCH_3), 4.53 (1H, s, CHAr), 6.18 (1H, d, *J*=8.8 Hz, ArH), 6.45 (1H, dd, *J*=7.2, 2.4 Hz, ArH), 6.77 (1H, d, *J*=8.4 Hz, ArH), 6.84 (2H, s, NH_2), 7.25 (2H, d, *J*=8.4 Hz, ArH), 7.83 (2H, d, *J*=9.2 Hz, ArH), 9.78 (1H, s, OH).

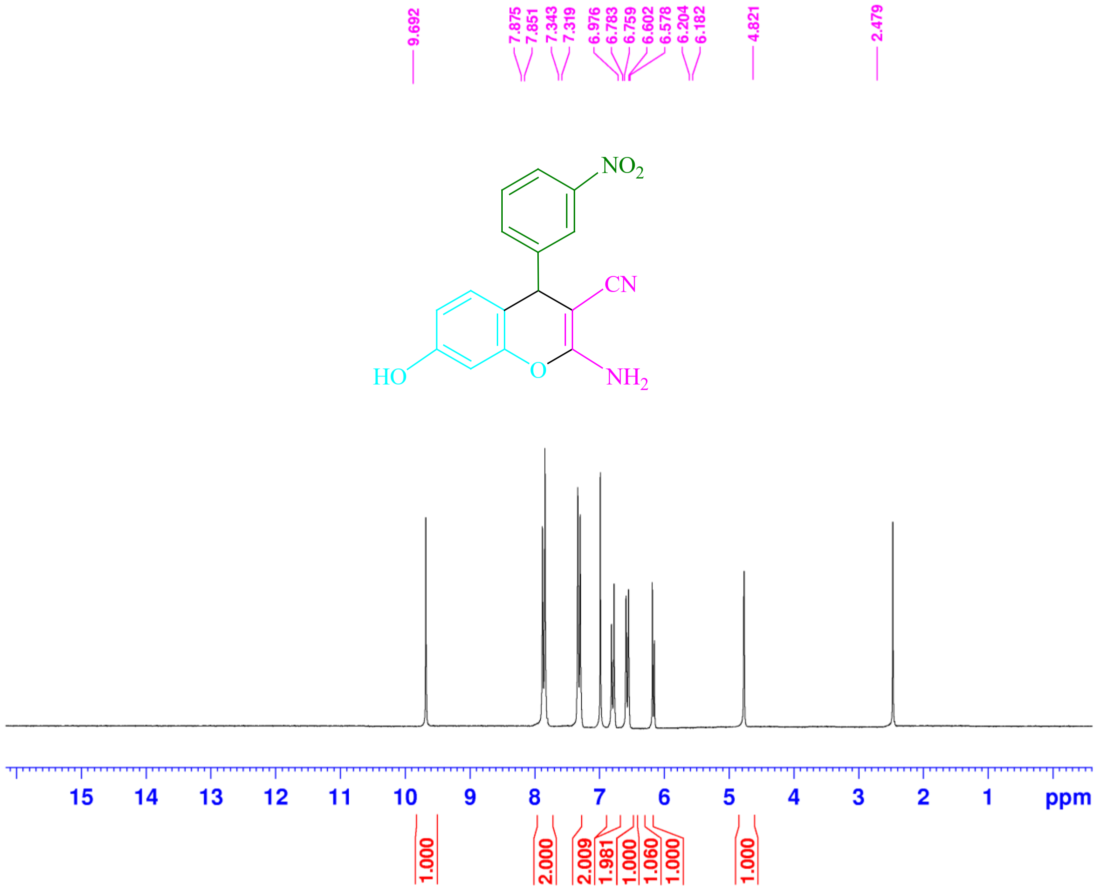

Figure 1. ^{1}HNMR Spectrum of compound (300 MHz, DMSO-d_6) of **4b**

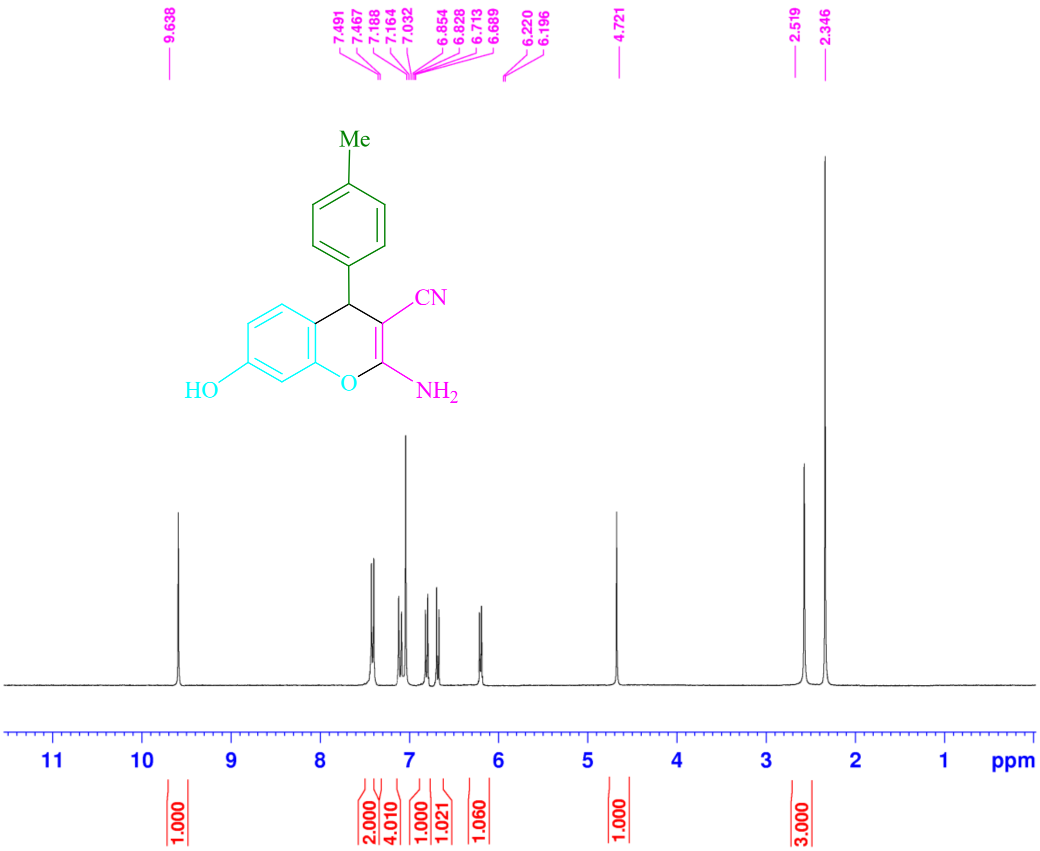

Figure 2. ^{1}HNMR Spectrum of compound (300 MHz, DMSO-d$_6$) of **4c**

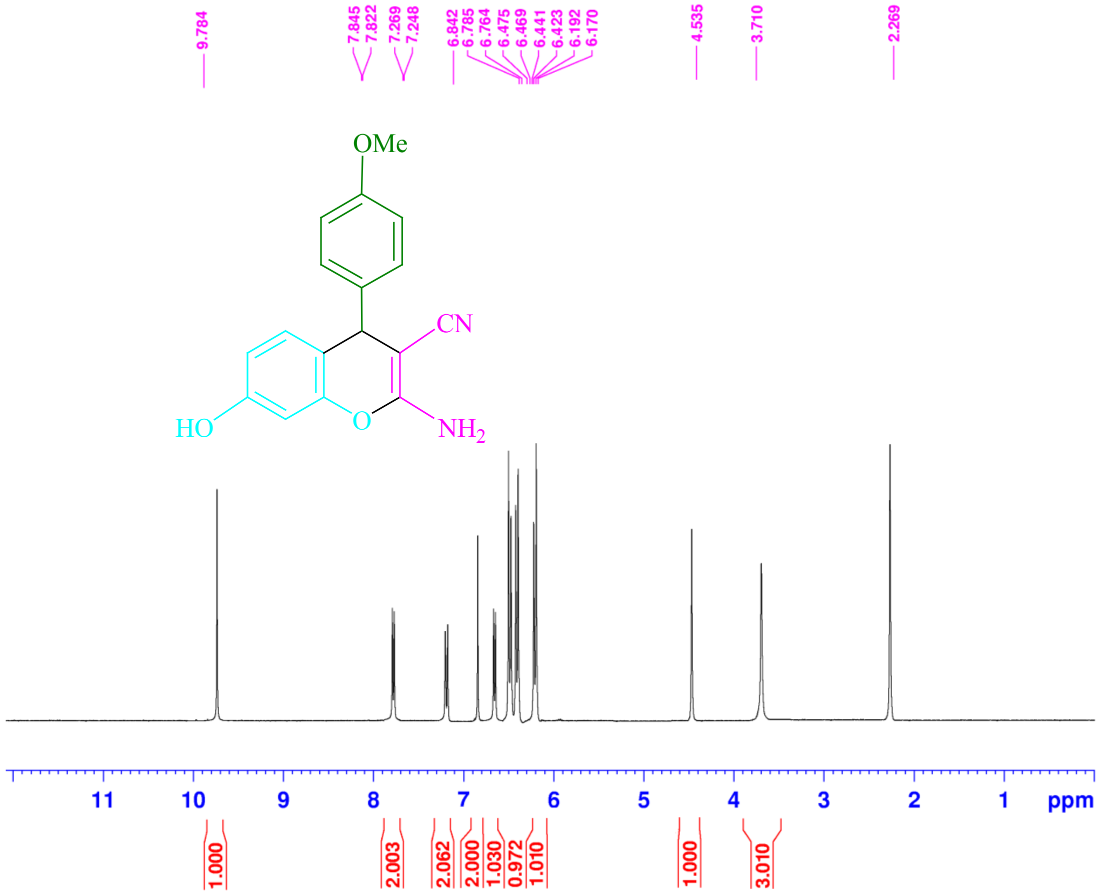

Figure 3. ^{1}HNMR Spectrum of compound (300 MHz, DMSO-d6) of **4p**
