# Supplementary material for: New Role for Photoexcited Na2 Eosin Y via the Direct Hydrogen Atom Transfer Process in Photochemical Visible-Light-Induced Synthesis of 2-Amino-4H-Chromene Scaffolds Under Air Atmosphere
Source: Front Chem. 2022 Jun 9;10:880257. doi: 10.3389/fchem.2022.880257 (PMC9218595; doi:10.3389/fchem.2022.880257)
Supplement: Supplementary file 2 [file DataSheet1.pdf]

Supplementary Material

1 Supplementary Data

2-Amino-3-cyano-7-hydroxy-4-(3-nitrophenyl)-4H-chromene (4b)

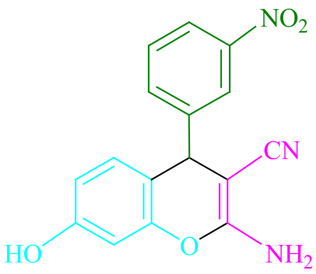

Yield: 94%; M.p. 166-168 °C; ^{1}HNMR (300 MHz, DMSO-d$_6$): 4.82 (1H, s, CHAr), 6.19 (1H, d, *J*=8.8 Hz, ArH), 6.59 (1H, d, *J*=9.6 Hz, ArH), 6.77 (1H, d, *J*=9.6 Hz, ArH), 6.97 (2H, s, NH_2), 7.33 (2H, d, *J*=9.6 Hz, ArH), 7.86 (2H, d, *J*=9.6 Hz, ArH), 9.69 (1H, s, OH).

2-Amino-3-cyano-7-hydroxy-4-(4-methylphenyl)-4H-chromene (4c)

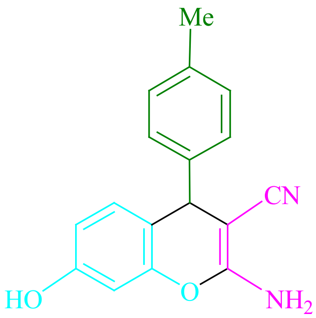

Yield: 91%; M.p. 185-187 °C; ^{1}HNMR (300 MHz, DMSO-d$_6$): 2.51 (3H, s, CH_3), 4.72 (1H, s, CHAr), 6.21 (1H, d, *J*=9.6 Hz, ArH), 6.70 (1H, d, *J*=9.6 Hz, ArH), 6.84 (1H, d, *J*=10.4 Hz, ArH), 7.03 (2H, s, NH_2), 7.17 (2H, d, *J*=9.6 Hz, ArH), 7.48 (2H, d, *J*=9.6 Hz, ArH), 9.63 (1H, s, OH).

2-Amino-3-cyano-7-hydroxy-4-(4-methoxyphenyl)-4H-chromene (4p)

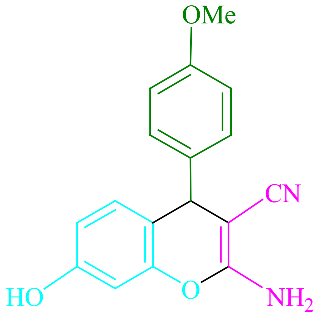

Yield: 88%; M.p. 208-210 °C; ^{1}HNMR (300 MHz, DMSO-d$_6$): 3.71 (3H, s, OCH_3), 4.53 (1H, s, CHAr), 6.18 (1H, d, *J*=8.8 Hz, ArH), 6.45 (1H, dd, *J*=7.2, 2.4 Hz, ArH), 6.77 (1H, d, *J*=8.4 Hz, ArH), 6.84 (2H, s, NH_2), 7.25 (2H, d, *J*=8.4 Hz, ArH), 7.83 (2H, d, *J*=9.2 Hz, ArH), 9.78 (1H, s, OH).

2 Supplementary Figures and Tables

2.1 Supplementary Figures

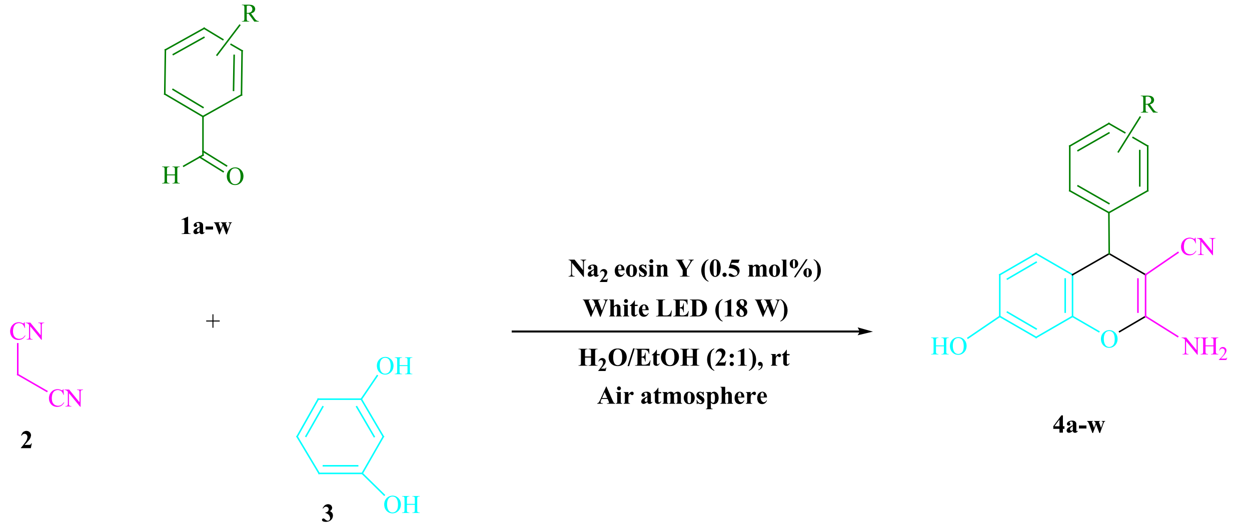

Supplementary Scheme 1.

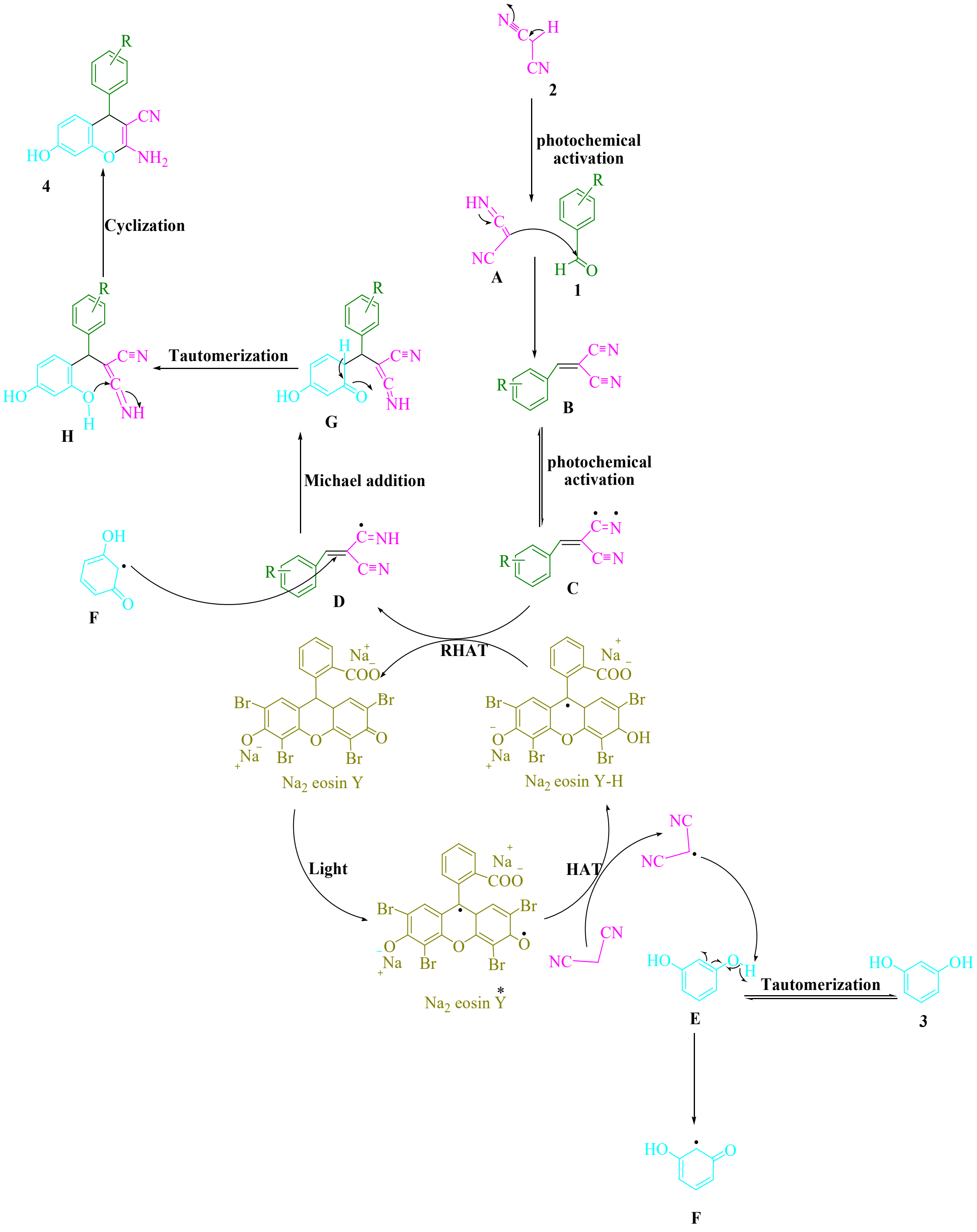

Supplementary Scheme 2.

2.2 Supplementary Tables

Table 1. Optimization table of photocatalyst, solvent and visible-light for the synthesis of **4a**[a]

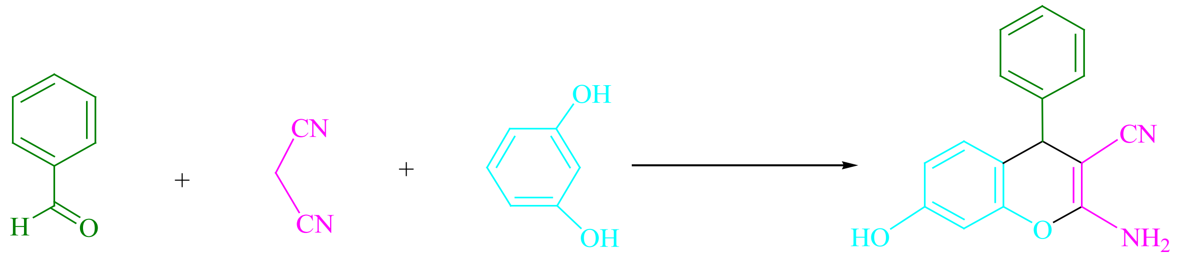

Entry	Photocatalyst	Light Source	Solvent (3 mL)	Time (min)	Isolated Yields (%)
1	—	White light (18 W)	H_2O/EtOH (2:1)	15	57
2	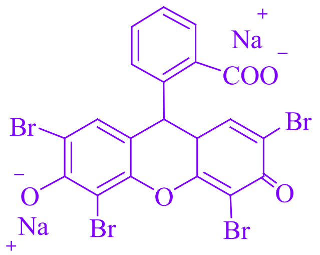 Na_2 eosin Y (0.2 mol%)	White light (18 W)	H_2O/EtOH (2:1)	5	78
**3**	**Na_2 eosin Y (0.5 mol%)**	**White light (18 W)**	**H_2O/EtOH (2:1)**	**5**	**93**
4	Na_2 eosin Y (1 mol%)	White light (18 W)	H_2O/EtOH (2:1)	5	93
5	Na_2 eosin Y (0.5 mol%)	White light (18 W)	EtOAc	5	67
6	Na_2 eosin Y (0.5 mol%)	White light (18 W)	EtOH	5	63
7	Na_2 eosin Y (0.5 mol%)	White light (18 W)	MeOH	10	52
8	Na_2 eosin Y (0.5 mol%)	White light (18 W)	H_2O	5	70
9	Na_2 eosin Y (0.5 mol%)	White light (18 W)	—	10	75

10	Na_2 eosin Y (0.5 mol%)	White light (18 W)	H_2O/EtOH (1:1)	5	79
11	Na_2 eosin Y (0.5 mol%)	White light (18 W)	H_2O/EtOH (1:2)	5	72
12	Na_2 eosin Y (0.5 mol%)	White light (18 W)	Toluene	20	46
13	Na_2 eosin Y (0.5 mol%)	White light (18 W)	$CHCl_3$	25	27
14	Na_2 eosin Y (0.5 mol%)	White light (18 W)	THF	25	32
15	Na_2 eosin Y (0.5 mol%)	White light (18 W)	CH_2Cl_2	25	30
16	Na_2 eosin Y (0.5 mol%)	White light (18 W)	DMSO	15	42
17	Na_2 eosin Y (0.5 mol%)	White light (18 W)	DMF	25	36
18	Na_2 eosin Y (0.5 mol%)	White light (18 W)	CH_3CN	10	49
19	Na_2 eosin Y (0.5 mol%)	Green light (18 W)	H_2O/EtOH (2:1)	5	81
20	Na_2 eosin Y (0.5 mol%)	Blue light (18 W)	H_2O/EtOH (2:1)	5	76
21	Na_2 eosin Y (0.5 mol%)	—	H_2O/EtOH (2:1)	20	<5
22	Na_2 eosin Y (0.5 mol%)	White light (10 W)	H_2O/EtOH (2:1)	5	71
23	Na_2 eosin Y (0.5 mol%)	White light (12 W)	H_2O/EtOH (2:1)	5	80
24	Na_2 eosin Y (0.5 mol%)	White light (20 W)	H_2O/EtOH (2:1)	5	93
25	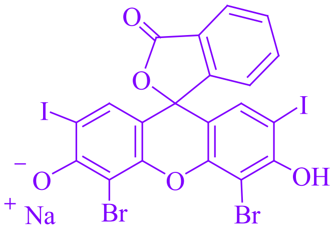 Erythrosin B (0.5 mol%)	White light (18 W)	H_2O/EtOH (2:1)	5	51
26	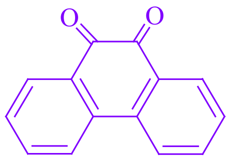	White light (18 W)	H_2O/EtOH (2:1)	5	53

	Phenanthrenequinone (0.5 mol%)				
27	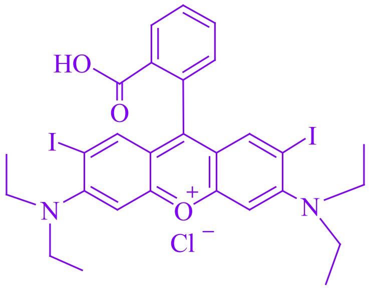 Rhodamine B (0.5 mol%)	White light (18 W)	H_2O/EtOH (2:1)	5	72
28	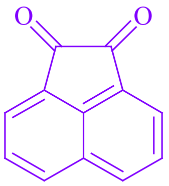 Acenaphthenequinone (0.5 mol%)	White light (18 W)	H_2O/EtOH (2:1)	5	57
29	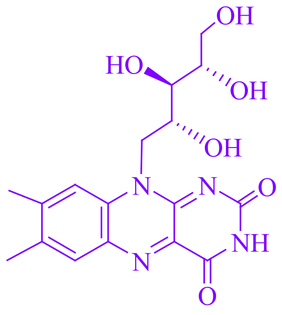 Riboflavin (0.5 mol%)	White light (18 W)	H_2O/EtOH (2:1)	5	64
30	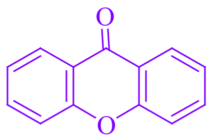 9*H*-Xanthen-9-one (0.5 mol%)	White light (18 W)	H_2O/EtOH (2:1)	5	60
31	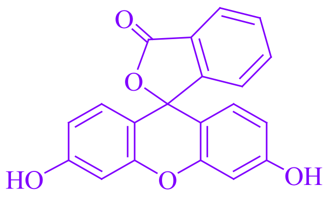	White light (18 W)	H_2O/EtOH (2:1)	5	77

	Fluorescein (0.5 mol%)				
32	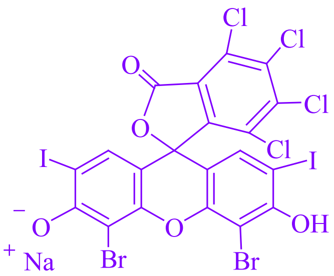 Rose bengal (0.5 mol%)	White light (18 W)	H_2O/EtOH (2:1)	5	68

[a]Reaction conditions: benzaldehyde (1 mmol), malononitrile (1 mmol), resorcinol (1 mmol) in visible-light, various solvents and photocatalysts at rt.

Supplementary Table 1.

Table 2. Photoexcited Na_2 eosin Y as photocatalyst for synthesis of 2-amino-4*H*-chromene scaffolds.

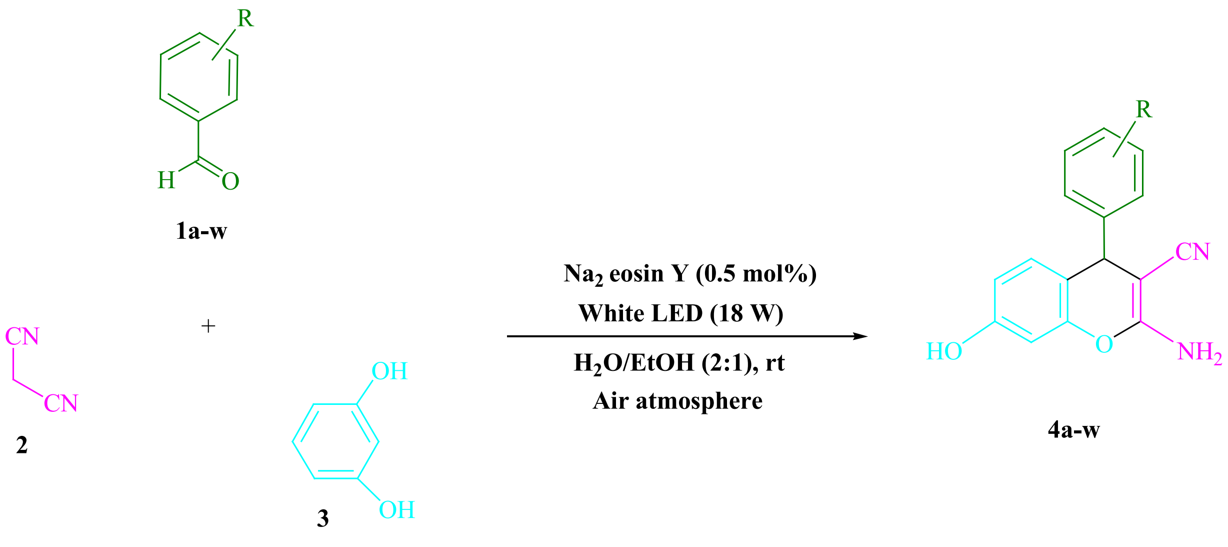

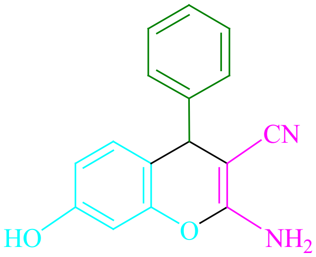<br>**4a** (5 min, 93%)<br>Mp. 234-236 °C<br>Lit. 232-234 °C [27]	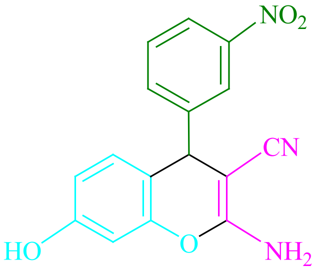<br>**4b** (5 min, 94%)<br>Mp. 166-168 °C<br>Lit. 168-170 °C [32]	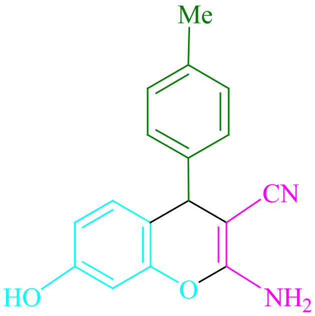<br>**4c** (3 min, 91%)<br>Mp. 185-187 °C<br>Lit. 186-188 °C [28]
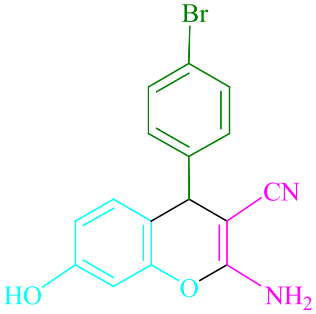<br>**4d** (10 min, 88%)<br>Mp. 223-225 °C<br>Lit. 222-224 °C [24]	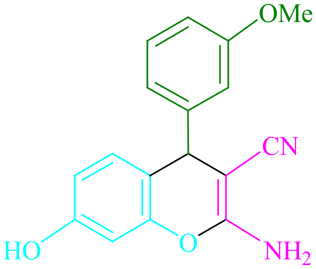<br>**4e** (7 min, 91%)<br>Mp. 179-181 °C<br>Lit. 180-182 °C [33]	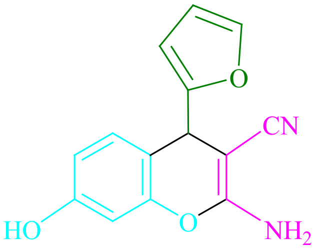<br>**4f** (5 min, 95%)<br>Mp. 192-194 °C<br>Lit. 190-192 °C [32]
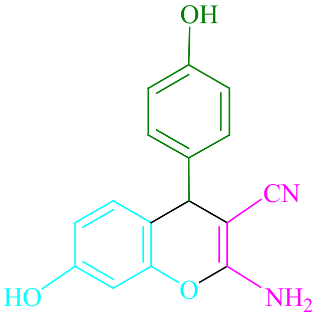<br>**4g** (10 min, 84%)<br>Mp. 249-251 °C<br>Lit. 250-252 °C [23]	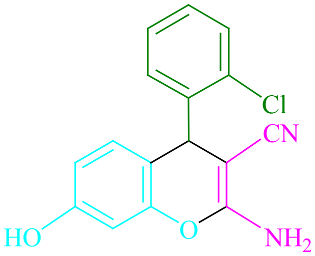<br>**4h** (5 min, 86%)<br>Mp. 187-189 °C<br>Lit. 189-191 °C [27]	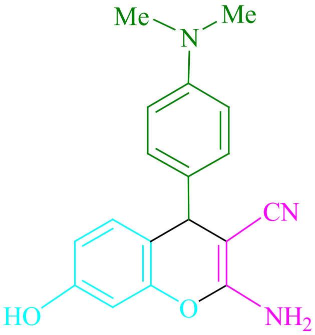<br>**4i** (5 min, 92 %)<br>Mp. 194-196 °C<br>Lit. 194-196 °C [24]

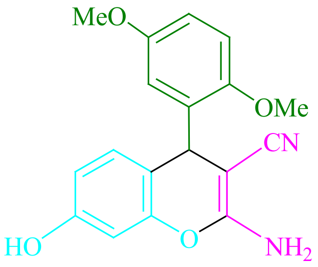

4j (9 min, 87%)
Mp. 200-202 °C
Lit. 198-200 °C [35]

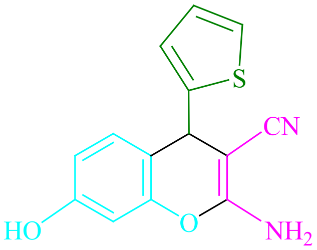

4k (5 min, 92%)
Mp. 211-213 °C
Lit. 210-212 °C [24]

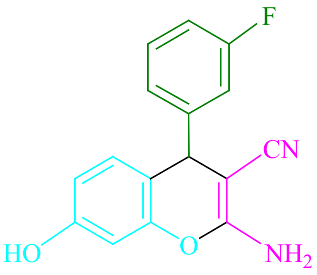

4l (3 min, 93%)
Mp. 146-148 °C
Lit. 148-150 °C [29]

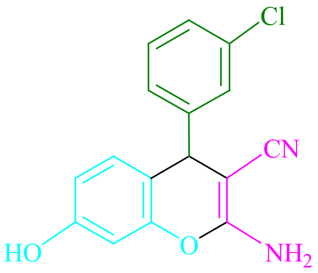

4m (7 min, 89%)
Mp. 175-177 °C
Lit. 176-178 °C [35]

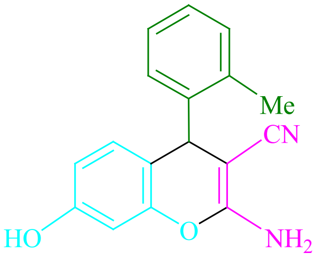

4n (3 min, 94%)
Mp. 227-229 °C
Lit. 228-231 °C [25]

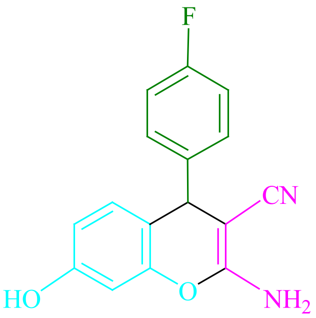

4o (3 min, 96%)
Mp. 189-191 °C
Lit. 188-190 °C [27]

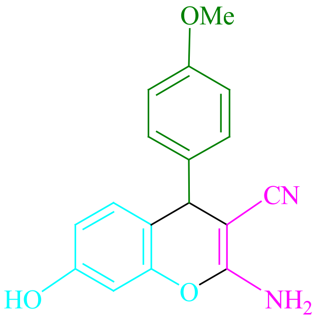

4p (7 min, 88%)
Mp. 208-210 °C
Lit. 210-212 °C [29]

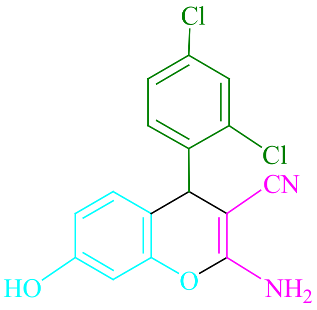

4q (10 min, 83%)
Mp. 259-261 °C
Lit. 257-259 °C [27]

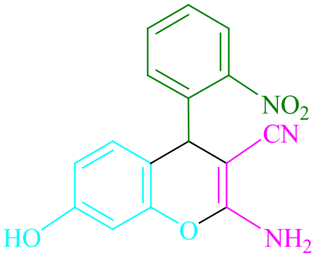

4r (3 min, 96%)
Mp. 160-162 °C
Lit. 162-163 °C [34]

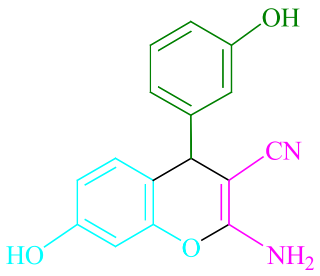<br>**4s** (8 min, 87%)<br>Mp. 218-220 °C<br>Lit. 219-221 °C [33]	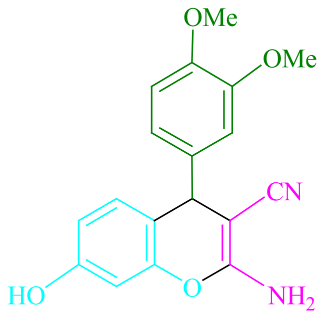<br>**4t** (9 min, 85%)<br>Mp. 229-231 °C<br>Lit. 227-229 °C [33]	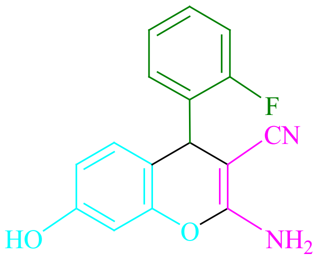<br>**4u** (3 min, 94%)<br>Mp. 201-203 °C<br>Lit. 200-202 °C [34]
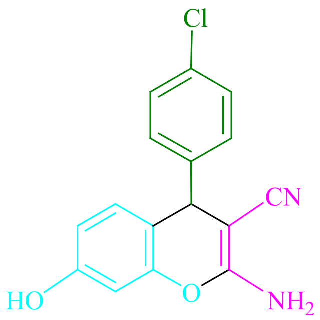<br>**4v** (7 min, 87%)<br>Mp. 163-165 °C<br>Lit. 162-163 °C [27]	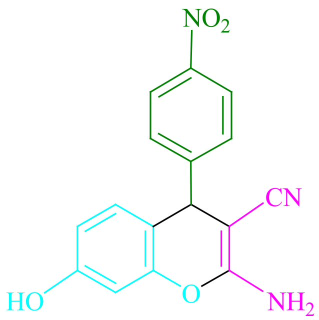<br>**4w** (5 min, 91%)<br>Mp. 167-169 °C<br>Lit. 166-168 °C [23]	

Supplementary Table 2.

Table 3. The comparison between the catalytic capacity of some catalysts in this work[a]

Entry	Catalyst	Conditions	Time/Yield (%)	References
1	glycine	H_2O, sonication	9 min/94	[22]
2	mesolite	EtOH, reflux	30 min/93	[23]
3	potassium phthalimide	H_2O, reflux	12 min/94	[24]

4	$MgFe_2O_4$NPs	EtOH, 65 °C	12 min/74	[25]
5	POM@Dy-PDA	EtOH/H_2O, reflux	15 min/95	[26]
6	P4VPy-CuI	H_2O, reflux	15 min/94	[27]
7	nanozeolite clinoptilolite	H_2O, reflux	15 min/92	[28]
8	WELFSA	H_2O, rt	1.5 h/88	[29]
9	tungstic acid functionalized SBA-15	H_2O, 100 °C	12 min/86	[30]
10	MIL-101(Cr)-SO_3H	H_2O, 100 °C	180 min/82	[31]
11	[$Et_2NH(CH_2)_2CO_2H$][AcO]	solvent-free, 60 °C	12 min/92	[32]
12	{[4,4′-BPyH][$C(CN)_3$]$_2$}	solvent-free, 80 °C	15 min/90	[33]
13	DBU	EtOH, MW, 50 °C	3 min/94	[34]
14	hydrotalcite	H_2O, 60 °C	4 h/95	[35]
**15**	**Na_2 eosin Y**	**visible light irradiation, H_2O/EtOH (2:1), rt**	**5 min/93**	**This work**

[a] Based on the three-component reaction of benzaldehyde, malononitrile and resorcinol.

Supplementary Table 3.
